# Private and Public Decisions in Social Dilemmas: Evidence from Children’s Behavior

**DOI:** 10.1371/journal.pone.0041568

**Published:** 2012-08-01

**Authors:** Daniel Houser, Natalia Montinari, Marco Piovesan

**Affiliations:** 1 Interdisciplinary Center for Economic Science, George Mason University, Fairfax, Virginia, United States of America; 2 Max Planck Institute for Economics, Jena, Germany; 3 Institute of Food and Resource Economics, University of Copenhagen, Copenhagen, Denmark; University of Maribor, Slovenia

## Abstract

Are selfish impulses less likely to be pursued when decisions are publicly observable? Is the presence of peers a potential solution to social dilemmas? In this paper we report data on the self-control decisions of children aged 6 to 11 who participated in games that require one to resist a selfish impulse for several minutes in order to benefit others. In Public Condition children make decisions in public view of the group of other participants, while in Private Condition they have the possibility to decide privately. We find that children aged 9 and higher are better able to resist selfish impulses in public environments. Younger children, however, display no such effect. Further, we find self-control substantially impacted by group size. When decisions are public, self-control is better in larger groups, while in private condition the opposite holds.

## Introduction

*Nothing makes it easier to resist temptation than a proper bringing-up, a sound set of values–and witnesses.*

–Franklin P. Jones.

Social dilemmas involve conflict between an individual’s short-term self-interest and a group’s ability to sustain social cooperation [1]. The temporal features of this conflict resemble individual self-control problems: succumbing to selfish temptations can detrimentally impact long-run individual interest [2], [3]. Moreover, in both contexts, even recognizing the long-term benefits does not prevent one from succumbing to selfish impulses. In view of these similarities, scholars in economics and psychology have recently investigated relationships between cooperation in social dilemmas and self-control [4], [5], [6].

Public environments enhance one’s ability to exercise self-control, and selfish temptations are more likely to be acted on under anonymity [7], [8]. To the best of our knowledge, however, this behavioral regularity has not been systematically explored as potential solution to social dilemmas. Our goal with this paper is to take a step in this direction. Doing this seems important, in part because it is often easier to control a person’s environment than to control their decisions.

This paper investigates the self-control decisions of children aged 6 to 11 in a social dilemma. Self-control in children is a topic that has received decades of scholarly attention [9]. In the environment we study, a group is made better off if all children within the group are able to avoid individual selfish temptations. Each child, however, is better off succumbing to temptation. We compare children’s ability to exercise self-control between conditions where their decisions are publicly observable to other group members and when they are not. The advantage to using children of these ages is that we are able to compare decisions of children aged nine years or younger to old children that are typically exhibit full theory of mind related skill [10], [11]. In doing this we are able to provide insight on the mechanism underlying any positive impact of public environments.

Our first hypothesis is that old children (aged 9 or older) will display increased self-control in public environments, while younger children will not. One reason is that older children, as a consequence of their ability to fully employ theory-of-mind reasoning, are more likely to believe that their group members will perceive them negatively if they succumb to a selfish temptation. The desire to avoid this feeling of “anticipated shame” is likely to be less pronounced in younger children. For instance, Ferguson et al [12] find that younger children -aged 7–9- associate shame with embarrassment, blushing, ridicule, and escape, while children age 9–11 additionally characterized shame as including more severe feelings such as feeling stupid, being incapable of doing things right, and not being able to look at others.

A second reason, again related to improved ability for theory-of-mind reasoning among older children, is that prosocial motives may provide an additional incentive to delay gratification. A positive relationship between a child’s age and their propensity for pro-social behavior has been documented in several studies [13], [14], [15].

In addition to our key comparison between public and private environments, within each condition we also vary the group size. Our second hypothesis is that group size impacts behavior differently in public than private decision contexts. In particular, when decisions are public behaving selfishly to many is “more shameful” than behaving selfishly to a smaller number. Consequently, larger groups in public decision environments should better deter selfishness. On the other hand, in private contexts one may be more concerned that other group members will be more likely to succumb to temptation. It follows that children in larger groups may be more likely to succumb to temptation more quickly, and this might be especially true of old children who are better able to engage in strategic reasoning.

## Procedures

We conducted the experiment during the period November 2010 - May 2011 in 22 classes (across 8 schools) in the district of Treviso (Italy). A total of 406 children aged between 6 and 11 years old participated in our study.

At the beginning of the experiment children received 5 colored bracelets for participating. These bracelets are in a transparent package and placed over each child desk. We told children that if *all of them* waited patiently for 10 minutes in silence and without touching, they would each receive 5 additional bracelets. However, if one child (or more) stops the time raising his/her hand, then only this child would receive the additional 5 bracelets, and the others would receive nothing beyond the initial 5 bracelets.

**Figure 1:**
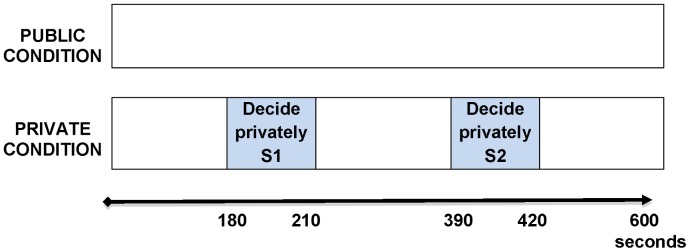
Timing of our two main conditions. In the Public Condition children make the decision to stop the time in view of all the other children. In the Private Condition, we gave children the additional possibility to stop the time privately within two periods of 30 seconds each (“S1” after 180 seconds and “S2” after 390 seconds). In these periods children could decide to stop the time privately using a specific report sheet previously distributed by the experimenter.

Our experiment has two main conditions (see Figure 1). In Public Condition children make the decision to stop the time in view of all the other children. In Private Condition, we gave children the additional possibility to stop the time privately using two slots of 30 seconds each (“S1” after 180 seconds and “S2” after 390 seconds). In these slots children could decide to stop the time privately using a specific report sheet previously distributed by the experimenter. In fact, before starting the game, the experimenters distributed two report sheets to each child. After 3 minutes each child can indicate if s/he wants to stop the time or continue with the game. The experimenters collected immediately all the report sheets and if none decided to stop the time, the experiment continued. After 3 minutes the experimenters repeated this procedure.

The game ended immediately when (at least) one child stopped the time, or otherwise after 10 minutes. At the end of the game, the experimenter distributed the bracelets: privately in the Private Condition and in front of the other classmates in the Public Condition. In both conditions, in case more than one child stopped the time we used a random device to determine who won the 5 additional bracelets.

The game we used is a common pool resource game (CPR). In a standard CPR environment [16] one wants to be sure not to “overfish” from a common pond, because doing so may entirely deplete the supply of fish. Thus, there is a tension between the desire to pull out more fish today, and the desire to have more fish tomorrow (the socially optimal outcome). Similarly, one can understand our game as including an initial small stock of bracelets. As long as nobody pulls these bracelets out too soon, they will multiply leaving enough for everybody. But, if somebody pulls them out, then the supply will be depleted and none will be available for others. This is the standard CPR tension.

Note that in a typical CPR environment the individual return to selfish behavior strictly dominates the return to cooperative behavior, despite the fact that the latter leads to the efficient outcome. In our CPR game a participant earns the same number of bracelets in both the selfish and cooperative outcomes (five additional bracelets in each case). Nevertheless, assuming there is a psychic cost to waiting, or that there is epsilon>0 probability of another taking the bracelets, then the strictly dominant outcome is for a participant to stop the game and take the bracelets as soon as they are able. Therefore, if a child decides to stop the time, he or she should stop the time immediately: if instead a child waits before acting selfishly, this is consistent with their use of self-control.

The design of our “private” condition reflects our concern that an entirely private decision environment would lead older children to stop the task immediately regardless of group size. Consequently, in order to increase the power of our design to detect group-size effects, we chose to include “public” decision phases in the “private” condition. The initial “public” decision phase can dissuade immediate stopping decisions, and perhaps build confidence that group members can resist stopping even in private phases. The manner in which such effects vary with group size informs our hypotheses of interest.

We are now in the position to specify in detail our research hypotheses:

1. Younger children (aged 6–9 years) will demonstrate less ability to wait than older children in both Conditions. It is well established in fact that the ability to delay gratification develops with age [17].
2. Older children (aged 10–11 years) will demonstrate a greater ability to wait in Public Condition. Older children may want to avoid the shame of appearing selfish, greedy or impatient, and be more likely to be motivated by prosociality, and therefore resist more in the Public Condition.
3. For older children, the effect of group size on the waiting time will be positive in Public Condition and negative in the Private Condition. The presence of an additional group member represents an additional threat to stop the game. Consequently, stopping the game quickly, before another can do so, becomes a more attractive option. This reasoning is offset in the Public Condition, however, by the fact that additional group member represents also an additional person to feel negatively towards the one who stopped the game. “Shame” may outweigh the “threat” effect, leaving stopping the game a less attractive option in larger groups.
4. We do not expect any difference in Public and Private Condition for younger children since they are not affected by the presence of others. At the same time we do not expect any effect of group size for young children.

In the following analysis we group children into “young” (first, second and third grade) and “old” (fourth and fifth grade) children. Our sample is balanced for age (48.4% are “young”) and gender (50.9% of boys). A set of χ^2^-tests confirm that is possible to compare children across these two conditions (Age Group: p  = 0.202; Gender: p  = 0.758). In addition, Table 1 and Table 2 report for each condition the percentage of young and old children by gender.

**Table 1:** Distribution of age groups and gender in Public Condition (in %).

	Public Condition (8 classes, N = 135)	
	Boys (N = 70)	Girls (N = 65)
Young (N = 60)	21.5%	23.0%
Old (N = 75)	30.4%	25.1%

**Table 2:** Distribution of age groups and gender in Private Condition (in %).

	Private Condition (8 classes, N = 142)	
	Boys (N = 71)	Girls (N = 71)
Young (N = 74)	24.0%	28.2%
Old (N = 68)	26.0%	21.8%

As previously mentioned, group size changes in each class: in Public Condition the average number of children in class is 19.3 (min = 15, max = 25) whereas in Private Condition is 15.9 (min = 12, max  = 20. This difference is slightly significant according to a two-sided Mann-Whitney test, p  = 0.055).

## Results

We present our data in two sections. First we analyze behavior at the group level, and then we focus on the behavior at the individual level.

### Waiting Time

**Figure 2:**
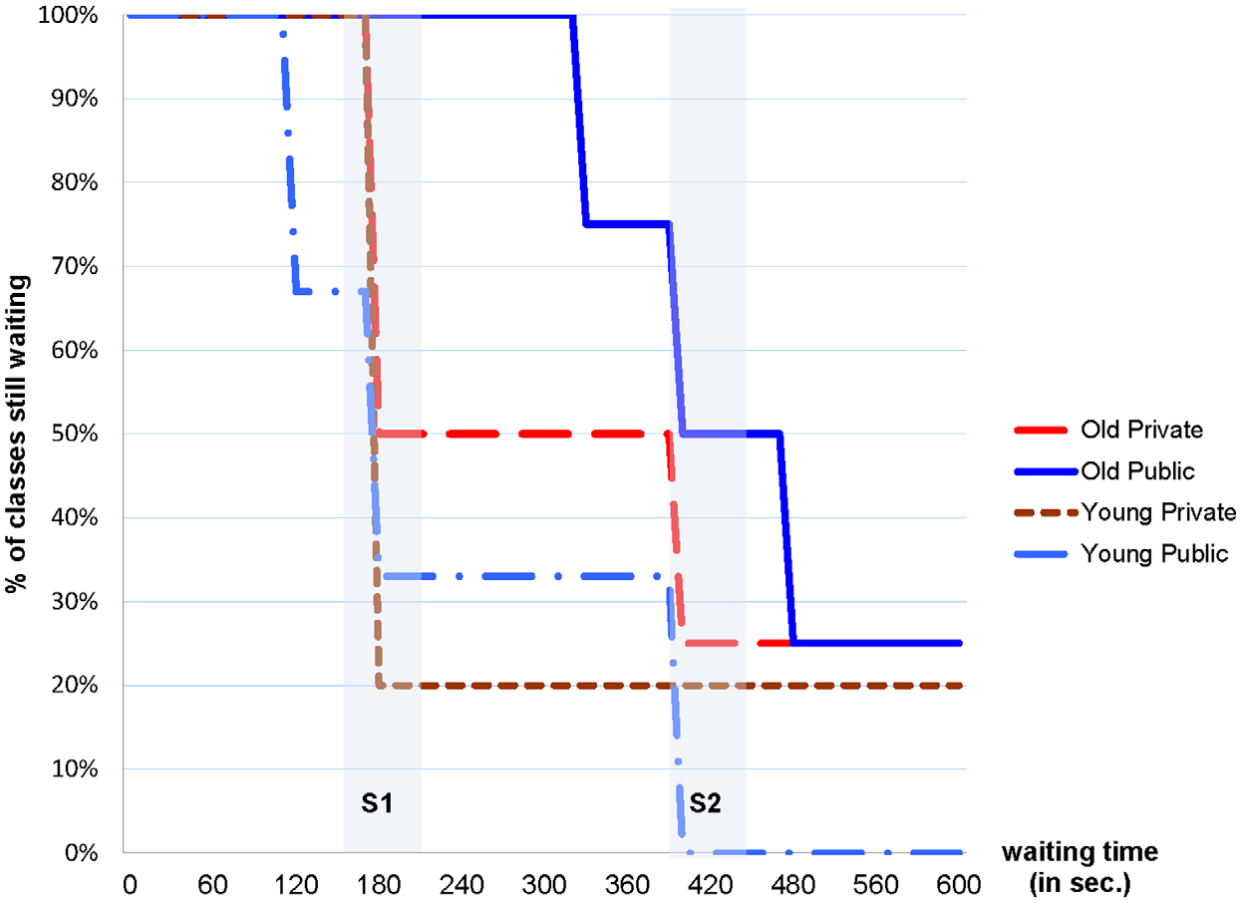
Time path of children’s ability to resist stopping the game. In both conditions, younger children stop sooner. Also, older children in Public resisted longer. All children that stopped the game in the Private Condition avoided doing so publicly.

The average waiting time in classes with young children is similar in our two Conditions (240 seconds in Public vs. 264 seconds in Private). Classes with older children (10–11 years of age) resist more, especially in Public Condition (457.5 seconds in Public vs. 337.5 in Private). Since we have a limited number of observations (16 classes) the only statistical difference is between the average waiting time of old and young children in the Public condition (two-sided Mann Whitney test p = 0.1084). Figure 2 shows the time path of children’s ability to resist stopping the game: in both conditions, younger children stop sooner. Also, older children in Public resist longer. Moreover, note that all children that stopped the game in the Private Condition used one of the two slots available for stopping the game in private, and thus in all cases they avoided stopping the game in view of others.

### Belief Elicitation

We asked other children (165 in total) -in other classes but of the same age- to guess the decisions of children in our sample. More specifically, we described the task in the Public or Private conditions and then asked children to guess whether, and how many, children eventually stopped the time. Correct guesses earned 10 bracelets (plus 2 bracelets for participating). We found that children correctly anticipated that in the Private condition a higher percentage of children stopped the time. Thus, children correctly anticipated that in the Private Condition the selfish decision is more likely than in the Public Condition, and therefore expected more children to stop the game. In addition, comparing the distributions of the guesses, we that children believe a smaller percentage of children will stop the game in Public (Kolmogorov-Smirnov, one sided, p-value = 0.082).

In summary, we have shown that groups of older children (aged 10–11 years) are able to wait longer before stopping the game. Second, older children display a greater ability to wait when they must stop the game in view of others. Third, children seem to hold correct beliefs about each other, in that they are able to predict the behavior of other children of their same age. In the next section, we study in greater detail the behavior of children at the individual level.

### Individual Behavior: Regression Analysis

Table 3 reports three logistic regressions where the dependent variable is a dummy variable taking value 1 if the child stopped the game, and 0 otherwise. As explanatory variables we include the condition (dummy Private equal to 1 if condition is private), group size, gender (dummy Male equal to 1 if the child is a boy) and age group (dummy Old equal to 1 if the child is enrolled in the 4th or 5th grade). We also control for some interactions between age group, group size, conditions and gender.

**Table 3:** Decision to stop the game.

	Dependent Variable Stop game ( = 1 if the child stops the game, 0 otherwise)		
	Logistic Regressions		Multilevel mixed-effects logistic regression
Model	(1)	(2)	(3)
Old	−2.26* (1.18)	−2.58* (1.36)	−1.07** (.48)
Private	2.35** (0.99)	−7.84*** (2.37)	−7.72** (3.41)
Group size	0.24** (0.12)	−0.07 (0.08)	−0.07 (0.13)
Male	0.41 (0.46)	0.43 (0.50)	1.08** (0.48)
Old*Male	1.99 (1.46)	2.05 (1.53)	–
Private*Group size	–	0.53*** (0.13)	0.52*** (0.18)
Constant	−8.06*** (2.78)	−1.55 (1.55)	−1.92 (2.61)
Observations	277	277	277
Wald chi2	10.38	66.55	25.51
Prob > chi2	0.0650	0.0000	0.001
Pseudo R-squared	0.1429	0.1933	–
Log pseudolikelihood	−73.93	−69.58	−71.31
Random effect parameter			
Private	–	–	4.60e−08 (.2222534)
Session	–	–	3.63e−09 (.3419029)

*Note: Dependent variable:* ***Stop game*** ( = 1 if the child stops the game, 0 otherwise). *Estimation methods: (1)–(2) Logistic regression. Estimation methods: (3)* Multilevel mixed-effects logistic regression. *Models 1–2: Robust Standard errors clustered on 16 groups, standard error in parenthesis; Models 3: Robust Standard errors clustered on 2 conditions, standard error in parenthesis.*

*** = significant at 1%; ** =  significant at 5%; * = significant at 10%.

Overall, our analyses provide compelling and robust evidence of significant effects of age, condition, and group size on the ability to resist stopping the game: i) age significantly reduces the probability of stopping the game; ii) being in Private Condition increases the probability of stopping the game; iii) group size has a positive effect -reducing the probability of stopping the game- in Public Condition, but a negative impact -increasing the probability of stopping the game in Private Condition.

More specifically, models (1)–(2) contain results from a set of Logistic regressions in which standard errors are clustered at the level of the group (our 16 classes). In model (1) we include as explanatory variables condition, age group, gender, group size and the interaction between age and gender. In model (2) we add to the specification of model (1) the interaction between group size and condition. These regressions show that being an “old” child reduces significantly the probability to stop the game both in models. Similarly, in both models, the condition variable Private is significant: its effect is positive in model (1) whereas is negative in model (2), once we introduce the interaction between Private and group size. Therefore, we find support that the joint effect of being in Private Condition and having an additional group member raises significantly the probability of stopping the game in comparison to the Public Condition. Model (1) reports evidence of a positive and significant effect of group size on the probability of stopping the game. Results in models (1) and (2) do not change if we cluster standard errors at the level of Condition.

Finally, in model (3) we report results from a multilevel mixed-effect logistic regressions with variance decomposed between conditions and classes. Model (3) suggests that males have a significantly higher stopping probability than females. Older children, instead, have a lower probability of stopping the game, as do children in the Private condition. Group size is insignificant, while the interaction between Private and group size has a positive and significant effect.

## General Discussion

In our experiment children aged 6 to 11 years participated in a novel social dilemma experiment. Our design adapts the “Marshmallow experiment” to groups: all children in a group receive a prize that doubles if they can all wait together for 10 minutes. However, if only one child fails to resist then only his or her prize doubles. We analyze behavior under two conditions: “Public” in which the decision to stop visible to all participants and “Private” in which the decision can be taken privately.

We find older children (aged 10–11 years) are in all cases more able to exercise self-control than younger children, in the sense that they are able to wait longer, on average, before acting selfishly. Moreover, older children are able to wait significantly longer when decisions are public than when they are private. Younger children (aged 6–9 years), on the other hand, demonstrate little difference between conditions. Further, we find the larger groups encourage self-control among older children in the public conditions, while this effect is absent among younger children and when decisions are made in private. We pointed out that children aged 10–11 have a more sophisticated understanding of the strategic interaction context as well as the way in which they are perceived by others. Consequently, older children are more sensitive to shame than younger children. This enables us to shed some light on reasons public environments inhibit selfish decision making. In particular, we argued the fact that public decision-making promotes pro-social behavior only in old children suggests it is due to a desire to avoid being perceived negatively by others. Our data seem specifically to rule out the possibility that increased self-control in the public condition are due to implicit threats of punishment or other forms of retaliation. The reason is that these would be expected to inhibit selfishness at all ages.

Our findings suggest that announcing decisions publicly and to large groups may be a step towards promoting cooperation in some social dilemma environments, especially those where delay of gratification plays an important role. More generally, the avenue towards promoting cooperation that we suggest requires intervention only at the level of the social decision environment. Such an approach might have distinct efficiency advantages over alternatives (e.g., monitoring and enforcing sanctions) that require costly interventions at the individual level [18].

Research on promoting cooperation using mechanisms that require intervention with monetary incentives at the individual level (e.g., punishment and reward) comprise the vast majority of the social dilemma literature. This paper takes a different tack by investigating whether cooperation can be promoted by changing the social decision environment in the absence of changes to monetary incentives. Our findings suggest that ensuring decisions are publicly available to large groups may indeed be an important part of a solution to some social dilemmas. In future research we intend to explore how monetary and social incentives might be combined in order to achieve increased pro-social decisions in charitable giving environments [19].
